# Assessing the Health Impacts of Contaminants on Green Turtles (*Chelonia mydas*) Along the Southern Coast of Brazil

**DOI:** 10.1002/tox.70064

**Published:** 2026-03-07

**Authors:** Camila Roberta da Silva Ribeiro, Andressa Maria Rorato Nascimento de Matos, Eloiza Teles Caldart, Liana Rosa, Camila Domit, Guilherme dos Santos Lima, Ana Paula Frederico Rodrigues Loureiro Bracarense

**Affiliations:** ^1^ Laboratory of Animal Pathology Universidade Estadual de Londrina Londrina Paraná Brazil; ^2^ Laboratory of Public Health and Zoonosis Universidade Estadual de Londrina Londrina Paraná Brazil; ^3^ Laboratory of Ecology and Conservation Center for Marine Studies, Universidade Federal do Paraná Pontal do Sul Paraná Brazil; ^4^ Environmental Studies Center (CEA) São Paulo State University (Unesp) Rio Claro SP Brazil

**Keywords:** ecotoxicology, environmental contamination, histopathology, PAH, sea turtles, trace elements

## Abstract

Chemical pollutants pose a significant threat to marine ecosystems. Among these contaminants are trace elements and polycyclic aromatic hydrocarbons (PAH). Green turtles can be employed to evaluate the oceans' health due to their coastal habitats and exposure to multiple threats. This study investigated the anatomopathological findings and assessed the levels of non‐essential elements and PAH in the organs of green turtles along the Paraná coast in the South of Brazil. The animals analyzed were found stranded between August 2015 and December 2019, by the Santos Basin Beach Monitoring Project team (PMP‐BS). Histopathological analyses were carried out, and the levels of Arsenic (As), Cadmium (Cd), Lead (Pb), and Mercury (Hg) and PAH (dry weight; mg/kg^−1^) were evaluated. The data were stored in the SIMBA database (Aquatic Biota Monitoring System). Simple and multiple logistic regression models were used to verify the possible interactions between histopathological findings and contaminant levels, and Spearman's correlation coefficient was employed to assess the correlation between non‐essential elements and the curved carapace length (CCL). A total of 86 specimens were analyzed. The main histopathological findings were systemic congestion and granulomatous infiltrate. Naphthalene was the most frequently observed PAH. Cd and As showed the highest levels, with Cd having more interactions with histopathological findings. Inflammation was observed in several organs and correlated with the presence of non‐essential elements (As, Cd, Hg, and Pb) and PAH. These results suggest a link between tissue changes in green turtles and environmental contaminants. However, further research is necessary to understand the extent and range of these effects.

## 1 Introduction

Sea turtles play a crucial role in marine ecosystems, interacting with a diverse range of species within an extensive and complex food chain [1, 2]. As long‐lived, and late sexual maturity animals, sea turtles are susceptible to the accumulation of contaminants through both ingestion and environmental exposure [3, 4]. For this reason, they are considered potential bioindicators for evaluating the health of ecosystems and the levels of marine pollution [5, 6, 7].

Environmental contamination is a significant problem affecting sea turtles, with pollutants such as trace elements and polycyclic aromatic hydrocarbons (PAH) being of particular concern. Trace elements are naturally from rocks and soil, entering the ocean through natural processes like erosion and lixiviation and soil erosion [8], but their chemical form and concentration can be affected by human activities such as industrial activities, mining, sewage disposal, and burning fossil fuels [9, 10].

Several studies have investigated trace elements levels in sea turtles in different regions of Brazil [8, 11, 12, 13, 14, 15, 16, 17]. Despite advancements in studies, a gap persists in understanding the relationship between trace element concentrations and animal health [12]. These trace elements have been linked to various health issues, such as gastrointestinal injuries, reproductive and growth complications, neurological disorders, and carcinogenic effects [12, 18, 19, 20].

Trace elements can be classified into essential and non‐essential categories. Non‐essential elements are not required for the metabolic functions and become toxic once their concentration exceeds a certain threshold [21]. Arsenic (As), cadmium (Cd), mercury (Hg), and lead (Pb) are considered non‐essential for sea turtles [22] and for them, low concentrations can be harmful to the organism [23].

Among these non‐essential elements, As, Cd, Pb, and Hg are regarded as particularly dangerous since they can induce different changes in health [24]. In studies involving loggerhead turtles ( *Caretta caretta* ), high hepatic concentrations of As have been linked to hepatic vacuolar degeneration and necrotizing hepatitis [25]. Freshwater turtles exposed to Cd exhibited dose‐dependent hepatocyte degeneration and necrosis [18]. Elevated blood Hg levels in *C. caretta* have been associated with reduced circulating lymphocyte counts and impaired B‐cell proliferation [26]. Conversely, no correlation has been found between blood levels of Cd, Pb, and As and the incidence of fibropapillomatosis in green turtles ( *Chelonia mydas* ) [27]. Although histopathological changes due to Pb exposure have not been documented in sea turtles, similar exposure in eagles has been linked to myocardial degeneration and necrosis, cerebral edema, and hemorrhage [28].

In addition to trace elements, another class of contaminants that impact marine organisms is PAH. PAH are considered ubiquitous organic contaminants that can be found in soil, air, sediments, and water [29]. They show widespread dispersion in the aquatic environment and varied toxicity [30]. Studies on the concentrations of PAH in *C. mydas* have been conducted in Brazil [31, 32] and in many other countries [5, 33, 34] most of them focusing on the detection and concentrations of these contaminants in different tissues. PAH, in general, can potentially cause developmental problems, egg fertilization, and hatching success [33, 35] and can also lead to carcinogenic issues [36]. Correlations between PAH and electrolyte balances were reported, suggesting that exposure to these environmental contaminants could affect the kidneys and salt glands in sea turtles [37].

The co‐occurrence of trace elements and PAH in marine environments introduces the possibility of synergistic toxicity, whereby their combined effects on marine organisms could be more harmful than the sum of their individual impacts. However, research into the simultaneous exposure to and co‐toxicity of organic and inorganic contaminants, as well as their combined adverse effects, remains limited [38, 39]. This underscores the need for further research exploring the potential combined impacts of these contaminants, providing an integrated view of environmental risks.

Most biomonitoring programs have focused on establishing baseline data for trace elements and PAH in blood and tissues. However, there remains a gap in our understanding of the histopathological changes associated with exposure to these contaminants, particularly in relation to the specific organs affected by PAH and non‐essential elements. While gross and microscopic analyses are commonly used in monitoring programs, investigating the connection between histological changes and exposure to environmental contaminants becomes essential for understanding their causes and effects on animals' health. Given the limited information on how these contaminants affect sea turtles, this study aimed to evaluate the histopathological findings in *C. mydas* found stranded on the Paraná coast and assess their relationship with levels of non‐essential trace elements and PAH in different organs.

## 2 Materials and Methods

### 2.1 Study Area

The study area is in the Paraná coastal area, in southern Brazil (Figure 1). This region, comprised of conservation units, encompasses significant marine and terrestrial environments [40]. It is recognized as a World Heritage Site for its exceptional biological and cultural diversity [41] and is vital for juvenile *C. mydas* , providing essential shelter, feeding grounds, and resting areas [42, 43].

**FIGURE 1:**
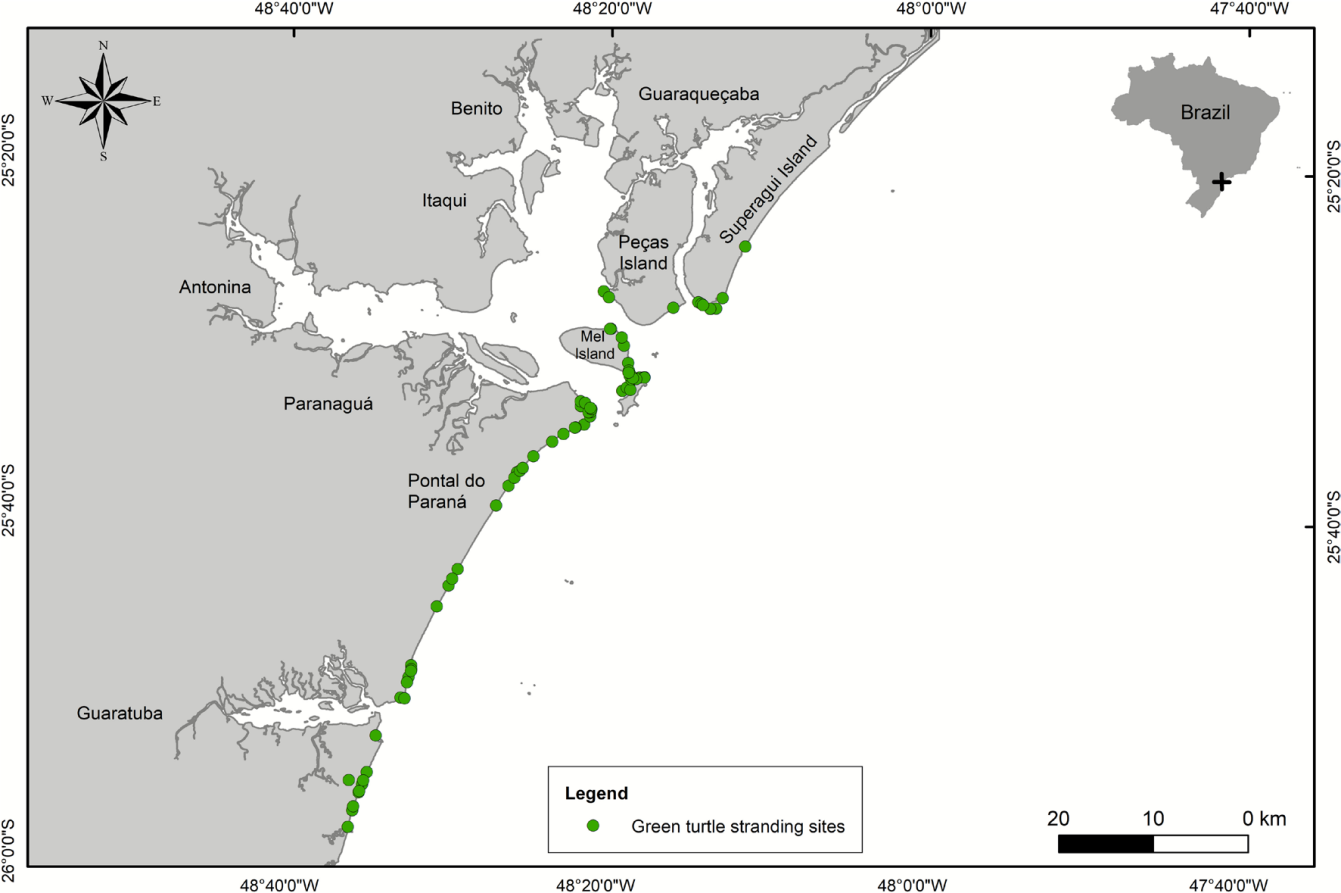
Coast of the State of Paraná, southern Brazil, showing the stranding locations of *Chelonia mydas* carcasses in fresh condition (*n* = 86) recorded between August 2015 and December 2019. *Source:* Laboratory of Animal Pathology (LAP), Universidade Estadual de Londrina (UEL).

The region experiences a subtropical climate, and its economy is primarily supported by tourism, agriculture, and fishing, as well as substantial port operations in Paranaguá and Antonina [44, 45].

Impacts arising from these economic activities have led to significant environmental degradation, primarily through pollution from chemical contaminants [46]. This pollution is attributed mainly to agriculture [47], port and industrial operations [48] and the discharge of domestic sewage [49].

### 2.2 Animals

The juvenile *C. mydas* analyzed were found stranded on the beach between August of 2015 and December of 2019 by the Santos Basin Beach Monitoring Project team (PMP‐BS). The PMP‐BS is a program required by the Brazilian Institute of Environment and Renewable Natural Resources (IBAMA) for the environmental licensing process for the exploration and flow of oil by Petrobras in the Pre‐Salt Complex in the Santos Basin.

The necropsies of the animals were carried out at the PMP‐BS base, located at the Center for Studies of the Sea (UFPR/Pontal do Paraná‐PR). Decomposition states of the carcasses were classified as “fresh” (organs with no visual alteration of color and texture), “moderate” (organs with some degree of color and texture alteration and a reduced presence of putrefaction gases), and “advanced” (organs with altered color and texture, affected by putrefaction gases) (adapted from [50, 51]). Each specimen had its curved carapace length (CCL) (cm) measured (mean ± SD), body condition classified as “poor,” “fair,” or “good” [52], and sex recorded. All data about the specimens were obtained through the SIMBA database (simba.petrobras.com.br/simba/web/). A total of 3640 *C. mydas* were found stranded; however, only individuals classified as being in a fresh state of decomposition were included in the present study.

### 2.3 Histopathological Analysis

The tissue samples, approximately 2 cm^3^, were fixed in a 10% buffered formalin solution. The samples were routinely processed, and histological slides were stained with hematoxylin and eosin and analyzed under an optical microscope. The organs analyzed included the lung, heart, spleen, liver, kidney, thymus, and thyroid. Fragments for histopathological analysis were taken only from carcasses in a fresh state. The histopathological results were stored in the SIMBA database [53].

### 2.4 Polycyclic Aromatic Hydrocarbons—PAH

The data about the levels of PAH in the liver of *C. mydas* were obtained from the SIMBA database [52]. The evaluation and interpretation of levels of PAH were carried out by the Laboratory of Marine Organic Chemistry at the Instituto Oceanográfico of the University of São Paulo (USP). The PAH evaluated included 16 priority PAH according to the U.S. Environmental Protection Agency (EPA): naphthalene, acenaphthylene, acenaphthene, fluorene, phenanthrene, anthracene, fluoranthene, pyrene, benzo(a)anthracene, chrysene, benzo(b)fluoranthene, benzo(k)fluoranthene, benzo(a)pyrene, indeno(1,2,3‐c,d)pyrene, dibenzo(a,h)anthracene, and benzo(ghi)perylene. Additionally, the compounds dibenzothiophene, perylene, and benzo(e)pyrene, as well as the following alkylated PAH, were evaluated: C1‐ to C4‐naphthalenes, C1‐ to C3‐fluorenes, C1‐ to C3‐dibenzothiophenes, C1‐ to C4‐phenanthrenes‐anthracenes, C1‐ and C2‐fluoranthenes‐pyrenes, and C1‐ and C2‐chrysenes.

The analytical procedures followed the recommendations of the EPA protocols: 3540C (extraction), 3610, 3630, and 3640 (purification), and 8270 and 8081B (quantification) [54, 55]. Quantification was based on internal standardization. The alkylated PAH were determined using the calibration curve of the non‐alkylated homologue. The detection limits of the method (LD) were defined as three times the standard deviation (SD) of the quantification of seven replicates of a sample (LD = 3 × SD) [56]. The limit of quantification of the method (LQ) was defined by the first point of the calibration curve. LD and LQ for each compound were calculated in relation to the extracted mass.

### 2.5 Trace Elements

The trace elements analyzed were As, Cd, Hg, and Pb (dry weight, mg/kg^−1^). The selection of the four non‐essential elements was based on the availability of an adequate number of specimens with existing analytical results within the PMP database. These elements (Cd, As, Pb, and Hg) were the most consistently analyzed across the sampled animals, allowing for a more robust and representative dataset. The evaluation and interpretation were carried out by the Environmental Studies Center of UNESP (CEA/UNESP). For determination of As, Cd, and Pb, samples were lyophilized following the method outlined [57]. The method consisted of initially weighing the sample (approximately 5 g) and subsequently introducing it frozen (approximately −80°C) into a freeze‐dryer (Alpha 1–2 LDplus—Christ). Freeze‐drying was carried out under the following conditions: temperature (−40°C) and pressure (0.11 mbar) for 72 h. After completion, the sample was re‐weighed to determine the moisture content, calculated as the difference between the initial and final mass. The dried sample was subsequently macerated and stored in a decontaminated plastic bottle (cleaned with 10% v/v HNO_3_) in a pressurized desiccator until digestion.

Sample digestion followed the EPA 3052 protocol using a microwave‐assisted sample digester (ETHOS UP—Milestone, Italy). In the first stage of the digestion procedure, the sample temperatures were raised to 170°C in 10 min, remaining at this temperature for 5 min [58]. In the second stage, the temperature was raised to 200°C in 3 min and kept constant for 20 min. The digested extract of the sample was quantitatively transferred to 50 mL centrifuge tubes (previously decontaminated with 10% V/V HNO_3_ and weighed). The digest was divided into three pseudo‐replicates from a new dilution, where 5 mL of the sample digest was transferred, and the volume adjusted to 15 mL with grade 1 ultrapure water; the dilutions were weighed using an analytical balance (mass of the concentrated digest and final mass of the pseudo‐replicates). Subsequently, the diluted digests (pseudo‐replicates) were analyzed by inductively coupled plasma mass spectrometry (ICP‐MS). Element determinations were based on EPA 6020A protocol (EPA 1998).

For Hg analysis, liver samples were directly digested without prior lyophilization [59]. Approximately 0.5 g of the fresh sample was digested in a microwave oven with an acidic mixture of double‐distilled aqua regia and subsequently analyzed in an Atomic Fluorescence Spectrometer (AFS) (PS Analytical model Millennium Merlin—Kent—UK). For the determination of Hg, the method used was based on EPA 245.7 and 7474 protocols. All values are available for public consultation in the SIMBA database [53].

### 2.6 Statistical Analysis

Excel software was used to tabulate data and perform descriptive analysis. Descriptive analysis included the mean, minimum, maximum, and standard deviation of trace elements and PAH. The independent variables analyzed in the study were the types of contaminants (trace elements/PAH), and the dependent variables were histopathological findings. Simple and multiple logistic regression models were performed with all possible combinations of dependent and independent variables. The significance level adopted was *p* < 0.05, and the association strength was measured using the odds ratio (OR) values and the respective 95% confidence interval. R environment version 3.6.2, along with the packages epitools [60] and epiDisplay [61] was used. Spearman's correlation coefficient was calculated to assess the correlation between the different trace elements and the CCL.

## 3 Results

### 3.1 Animals

A total of 86 specimens of *C. mydas* were evaluated. The average CCL was 36.0 ± 5.1 cm (± SD) (ranging from 23.2 to 51.3 cm), and the body mass was 5.38 ± 2.18 kg (± SD) (ranging from 1.72 to 13.8 kg). The visual body condition was assessed as “poor” in 10 animals, “fair” in 22 animals, and “good” in 54 animals. Regarding the sex of the individuals, 72 were females, 12 were males, and the sex of two animals was undetermined.

### 3.2 Histopathological Analysis

The liver and lungs showed a higher frequency of histological changes. In the liver, the most common alterations were hepatocyte cytoplasmic vacuolation (hepatic degeneration) (84.7%) and congestion (40.0%) (Figure 2A,B). In the lungs, congestion (50.6%), hemorrhage (30.4%), and granulomatous infiltrate (29.1%) were the predominant findings (Figure 2C). Congestion was the most prevalent alteration observed across all organs (Table 1). Granulomatous inflammation was frequently observed in various organs, and an association with intralesional parasites (trematodes) was noted in the lung (10/24 cases; 41.6%, Figure 2D) and spleen (12/13 cases; 92.3%). Additionally, edema was a common alteration in the lung (19 out of 83 cases; 22.9%), and heterophilic splenitis was observed in the spleen (12 out of 82 cases; 14.6%).

**FIGURE 2:**
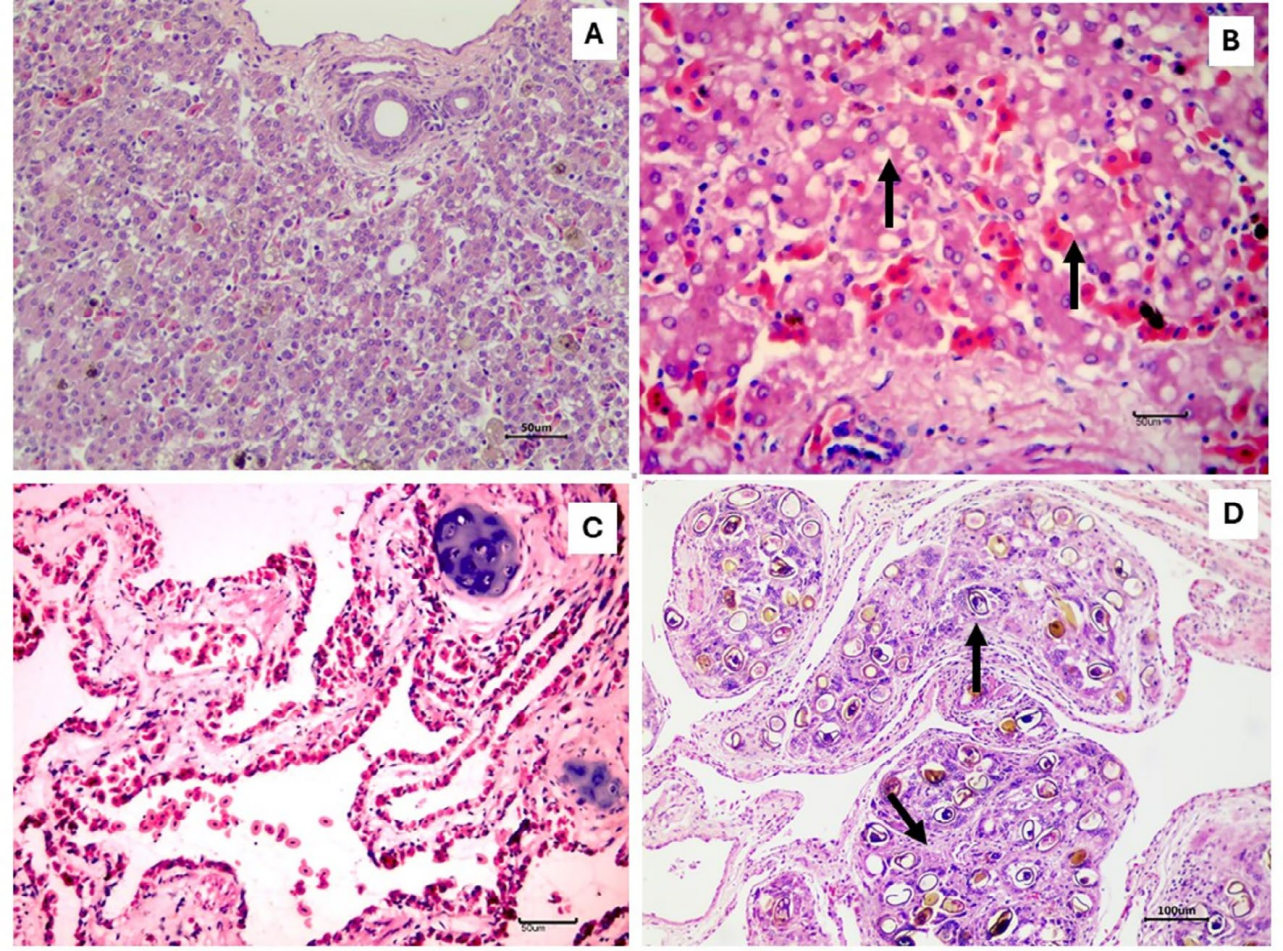
Histopathological changes in the liver and lung of *Chelonia mydas* found stranded on the Paraná coast. (A) Liver showing mild hepatocyte degeneration. Scale bar 50 μm. (B) Hepatocytes showing cytoplasmic vacuolation (arrows) and sinusoidal congestion. Scale bar 50 μm. (C) Lung showing moderate faveolar congestion. Scale bar 50 μm. (D) Granulomatous pneumonia with intralesional trematode eggs (arrows). Scale bar 100 μm. (A–D) Hematoxylin–eosin staining.

**TABLE 1:** Histopathological findings in organs of *C. mydas* found stranded on the Paraná coast, southern Brazil, between 2015 and 2019 (*n* = number of individuals analyzed).

	No changes	Congestion	Hemorrhage	Degeneration	Necrosis	Lymphocytic infiltrate	Histiocytic infiltrate
Lung *n* = 83	6 (7.2%)	42 (50.6%)	24 (30.4%)	—	—	6 (7.6%)	24 (28.9%)
Liver *n* = 85	1 (1.1%)	34 (40.0%)	—	72 (84.7%)	—	—	13 (15.2%)
Spleen *n* = 82	23 (28.4%)	27 (32.9%)	—	13 (15.8%)	1 (1.21%)	—	13 (15.8%)
Kidney *n* = 82	12 (14.6%)	25 (30.4%)	8 (9.75%)	10 (12.2%)	8 (9.75%)	12 (14.6%)	10 (12.2%)
Thymus *n* = 68	25 (36.7%)	21 (30.8%)	4 (5.8%)	8 (11.7%)	—	—	4 (5.8%)
Thyroid *n* = 59	24 (40.6%)	26 (44.6%)	11 (18.6%)	5 (8.47%)	—	1 (1.69%)	3 (5.08%)

### 3.3 Polycyclic Aromatic Hydrocarbons

The levels of PAH were evaluated in the liver tissue of 76 animals. PAH were detected in 39.5% of *C. mydas* specimens, and the detectable levels are shown in Table 2. Naphthalene was the main PAH observed (3.09 mg/kg^−1^ ± 11.8), followed by phenanthrene and fluoranthene.

**TABLE 2:** Levels of polycyclic aromatic hydrocarbons (dry weight; mg/kg) registered in the liver of *C. mydas* found stranded on the Paraná coast, southern Brazil, between 2015 and 2019.

	Minimum	Maximum	Mean	Standard deviation	*N* > LD^a^	∑
Acenaphthene	> LD	0.53	0.07	0.60	1 (1.31%)	5.25
Acenaphthylene	> LD	6.24	0.14	0.14	2 (2.63%)	11.0
Benzo(a)anthracene	> LD	0.65	0.09	0.74	1 (1.31%)	0.65
Benzo(b)flourantene	> LD	0.62	0.08	0.71	1 (1.31%)	0.62
Chrysene	> LD	0.67	0.09	0.77	1 (1.31%)	0.67
Phenanthrene	> LD	13.3	0.32	1.83	5 (6.50%)	24.0
Fluoranthene	> LD	5.57	0.16	0.77	5 (6.50%)	12.4
Fluorene	> LD	0.64	0.02	0.09	2 (2.63%)	1.12
Naphthalene	> LD	81.2	3.09	11.8	29 (38.1%)	234
Pyrene	> LD	2.21	0.61	0.32	3 (3.94%)	4.67

^a^ LD—Limit of detection is the smallest amount analyzed in a sample that can be detected.

### 3.4 Non‐Essential Elements

The presence of non‐essential element levels was evaluated in 79 animals. Some specimens presented extremely high levels of contaminants, which interfered with the statistical results; therefore, even though these results need to be biologically interpreted, they were removed from the statistical analyses (As—9.600; 6.621; Cd—17.15; 21.02; Hg—1.126; 1.787; 2.862; Pb—1.202). Regarding the concentrations of the trace elements, it was observed that Cd > As > Hg > Pb. The levels (mean ± SD) of these elements are shown in Table 3.

**TABLE 3:** Levels of arsenic, cadmium, mercury, and lead (dry weight, mg/kg) in the liver of *Chelonia mydas* found stranded on the Paraná coast, southern Brazil, between 2015 and 2019.

	Min.	Max.	Mean	SD	*N*
As	1.81	74.7	10.3	±10.2	77
Cd	3.58	78.6	19.1	±14.2	77
Hg	0.17	10.0	0.83	±1.17	76
Pb	0.04	19.4	0.64	±2.22	78

Abbreviations: As, arsenic; Cd, cadmium; Hg, mercury; Min., minimum; Max., maximum; Pb, lead; SD, standard deviation.

### 3.5 Histological Changes and Contaminants

A simple logistic regression model was employed to verify the relationship between histopathological changes and the presence of contaminants. We observed a significant relation to some contaminants and granulomatous inflammation (Cd in spleen, OR—1.07, *p* = 0.003 and lung OR—1.04, *p* = 0.028; Pb in lung—OR—6.24, *p* = 0.020; and PAH in liver OR—15.2, *p* = 0.013) (Table 4). There was a positively significant correlation between the CCL and Cd (*p* = 0.02; rho = 0.25), as well as CCL and Pb (*p* = 0.007; rho = 0.30). The concentration of some contaminants also had a significant positive correlation: Hg and As (*p* = 0.003; rho = 0.50), Pb and Cd (*p* = 0.0002; rho = 0.55).

**TABLE 4:** **—**Pathological findings significantly associated with contaminants in *Chelonia mydas* from the Paraná coast, southern Brazil.

Contaminants	Lesion	Coefficient	Standard error	Degrees of freedom	*p* Wald	OR (95% CI)
Cadmium	Hemosiderosis	0.04026	0.01879	73	0.032	1.04 (1.00–1.08)
Cadmium	Granulomatous splenitis	0.06861	0.02302	71	0.002	1.07 (1.02–1.12)
Cadmium	Granulomatous pneumonia	0.04126	0.01878	72	0.028	1.04 (1.01–1.08)
Lead	Granulomatous pneumonia	1.8304	0.7842	73	0.020	6.24 (1.34–29.00)
PAH	Granulomatous hepatitis	2.726	1.093	71	0.013	15.2 (1.70–130.00)

Abbreviation: PAH, polycyclic aromatic hydrocarbons.

## 4 Discussion

There is a growing interest in studies on marine environmental pollution in recent years, but there is still much to be explored about how contaminants can affect the health of marine fauna, especially sea turtles. To the author's best knowledge, no previous research in Brazil has assessed histopathological changes and attempted to statistically correlate them with the contaminants in *C mydas*. In this study, the presence of Cd, Pb, and PAH was associated with a significant inflammatory response.

The present study predominantly identified juveniles and females, with most specimens exhibiting good body condition. A high proportion of females in the population relative to males has also been observed in other studies [6, 62]. This study evaluated juvenile *C. mydas* , which are characterized by omnivorous feeding habits. According to previous studies, this dietary pattern may influence tissue concentrations of trace elements. Juveniles are more likely to ingest prey items with higher trace element loads compared to adults, which exhibit predominantly herbivorous feeding behavior [15, 17, 63].

The liver and lungs exhibited a greater frequency of histological changes. In the lungs, congestion and hemorrhage were the main findings, while in the liver hydropic degeneration and congestion were more frequent. In other tissues, congestion was the main finding, indicating a systemic process. These findings have been associated with hypoxia/asphyxia caused by drowning, a common cause of death worldwide in sea turtles [64, 65]. Recently, similar findings were reported in *C. caretta* showing bycatch interaction [66].

Granulomatous infiltrate was a common finding in all organs, and the inflammatory infiltrate associated with trematodes of the Spirorchiidae family was frequently observed in other studies [67, 68]. Trematodes of this family inhabit the blood circulation, where they copulate, and the eggs migrate to other organs, generating a systemic inflammatory response [69]. In the present study, severe parasitic granulomatous inflammation was more prevalent in the lungs and spleen, consistent with findings from previous studies [70, 71]. These changes lead to a loss of respiratory function due to the thickening of the interfaveolar septa, which compresses the air spaces. Additionally, changes in immunity are expected, as the lymphoid tissue in the spleen becomes depleted.

Sea turtles can be susceptible to contaminants in different ways. The urbanization of coastal areas increases anthropogenic impacts including pollutants sources and other stressors that affect the overall quality of the ecosystem [72]. PAH are known as persistent pollutants, with great capacity to produce metabolites, widely dispersed across the globe, capable of being transported over long distances, and causing serious health impacts on animals and humans [73]. In this study, the main PAH observed was naphthalene, present in almost 40% of specimens, followed by phenanthrene and fluoranthene, each observed in a small number of individuals (both 6.6%). Naphthalene, a PAH derived from tar and petroleum, is also a byproduct of burning wood or tobacco [74].

A previous study in the Northeast region of Brazil found naphthalene in 82.3% of analyzed sea turtles, while phenanthrene and fluoranthene were detected in 100% of the samples; however, the average concentration of naphthalene in that study was lower than what we found (67.9 vs. 3.09 mg/kg^−1^, d.w.) [32]. Despite these results, differences in analytical methods, particularly in the sample purification processes, may also relate to the concentration variations observed between regions. Future studies could address these discrepancies to better inform regional mitigation strategies. Similarly, naphthalene was one of the most frequently detected PAHs in blood of juvenile *C. mydas* (0.493 mg/kg^−1^) in the Gulf of Oman [33] reinforcing the importance of strengthening efforts in monitoring this pollutant and its potential effects on marine life.

Sea turtles can absorb PAH through the skin, mucous membranes, eggshells, inhalation, and ingestion of contaminated prey [75]. In this study, animals exposed to PAH showed a positive association to granulomatous hepatitis. PAH are metabolized in the liver by cytochrome P450 (CYP) enzymes and can induce oxidative stress [33]. Consequently, it can be hypothesized that the oxidative stress response resulting from PAHs detoxification contributes to establishing the inflammatory process. The occurrence of granulomatous hepatitis in sea turtles exposed to PAH indicates a chronic hepatic inflammatory response consistent with contaminant‐induced toxicity, reflecting environmental pollution and potential impacts on health and fitness.

Additionally, in this study, Cd, originating from agricultural and industrial waste [76], exhibited the highest mean concentration of trace elements (19.1 mg/kg^−1^). These findings are comparable to those reported in other studies on *C. mydas* in Brazil (18.8 mg/kg^−1^; 25 mg/kg^−1^) [15, 17] and Mexico (16.9 mg/kg) [77]. Research suggests that the Cd levels detected in juvenile *C. mydas* may be linked to the predation of zooplankton and other microorganisms in the South Atlantic Ocean [8].

Interestingly, this study found a significant association between Cd and granulomatous pneumonia, granulomatous splenitis, and hemosiderosis. In a study with rodents exposed to cadmium, alveolar macrophages became sources of inflammatory mediators, contributing to acute and chronic lung injury, while their maturation was impaired, resulting in an ineffective immune response; additionally, systemically, cadmium reduced spleen cell viability and induced apoptosis in splenocytes, promoting immunosuppression [78]. Given that the presence of contaminants in marine turtle organisms can induce immunosuppression and consequently increase susceptibility to infectious agents, it is plausible to hypothesize that Cd exposure may compromise the immune response of *C. mydas* , potentially increasing the vulnerability of these turtles to trematode parasitic infections in the lungs and spleen [79].

The second highest trace element detected in the present study was As (10.2 mg/kg), which can originate from both natural and anthropogenic sources, and its abundance is mainly attributed to ore smelting, coal burning, and industrial waste [80, 81]. This concentration is like a previous study conducted on the southeastern coast of Brazil, including the samples from the Paraná coast (9.0 mg/kg) [17]. However, both values are significantly higher than those detected in previous studies conducted with *C. mydas* that reported in liver levels of 1.2 μg/g [82] and varying from 0.87 to 5.3 μg/g [83]. Arsenic is considered one of the most harmful elements [84] and is known to be hepatotoxic, causing inflammation through increased production of proinflammatory cytokines [85, 86]. In a study involving *C. caretta* stranded in the Canary Islands, Spain, animals with the highest levels of As exhibited vacuolar hepatic degeneration and necrotizing hepatitis [25].

In the present study, the concentration level of Cd showed a significant association with hemosiderosis, a condition characterized by the accumulation of iron [87, 88]. Cd can induce hemolytic anemia, which releases iron and leads to hemosiderosis [89]. However, it was not possible to evaluate anemia in the specimens analyzed, which represents a limitation of this study and a scientific recommendation for future studies.

The average concentration of Pb and Hg had the lowest mean values compared to the other elements. However, even in low concentrations, these elements can cause serious health effects. Intriguingly, our findings revealed a significant association between lead exposure and granulomatous pneumonia. Furthermore, exposure to lead elevated the odds of animals developing granulomatous pneumonia by 6.24 times. It is plausible that lead‐induced lung toxicity exacerbated the pulmonary lesions caused by trematodes. While there is a lack of data regarding pulmonary findings and lead exposure to green turtles, studies involving rats exposed to varying concentrations of lead demonstrated severe interstitial pneumonia, fibrosis, and alveolar collapse induced by inflammatory and apoptotic changes [90].

Regarding the registered lead content in this study (0.64 mg/kg^−1^), a similar result was observed in a broader survey in southeastern Brazil (0.75 mg/kg^−1^) [17]. In other studies, higher Pb levels were found in *C. caretta* from the Mediterranean coast (6.12 mg/kg^−1^) [91] and in loggerhead turtles in Turkey (2.73 mg/kg^−1^) [92]. It is important to note that *C. mydas* and *C. caretta* occupy distinct foraging habitats, and the levels of trace elements in turtle tissues may correlate with their dietary habits in various regions [92].

In addition to specific levels from various trace elements, Cd and Pb demonstrated a correlation with the size of sea turtles (CCL), suggesting a potential bioaccumulation process for these elements. Since our findings focus on juvenile females, these results highlight the long‐term impacts of these contaminants on individuals and populations, as they can influence reproductive parameters.

The mean Hg concentration in this study was 0.83 mg/kg^−1^, like another study conducted with *C. mydas* on the Ceará coast in northeastern Brazil (0.53 ± 0.26 mg/kg^−1^) [93]. Conversely, green turtles from Santos Basin, Brazil showed a higher mean concentration (2.00 ± 22.0 mg/kg^−1^) [17]. The Santos Basin presents areas exposed to greater influence of human activities than the Paraná coast, which probably leads to higher Hg levels. These higher concentrations are found in regions where domestic and industrial wastewater and petroleum activities are discharged [94], such as the Santos and São Vicente estuarine complex (SP) [95].

A limitation for a better understanding of the effects of these elements on the health of sea turtles is the absence of regulation of maximum tolerable levels for these elements with potential toxicity. In Brazil, legislation establishes maximum tolerable levels of contaminants (As −1.00; Cd 0.05; Hg 0.50 and Pb 0.30 mg/kg^−1^) in fish (raw, frozen or chilled) intended for human consumption [96]. When comparing these values with those found in the liver of the animals in this study, highly elevated levels can be observed, mainly of Cd and As (19.1 and 10.2 mg/kg, respectively). Consequently, further research is required in order to evaluate the toxicity levels of these elements in *C. mydas* , as well as the impact of exposure to various contaminants and their cumulative effects on sea turtles, individually and for their habitat.

## 5 Conclusion

There is limited data on contaminant‐related histopathological alterations and health conditions in *C. mydas* . In this study, we identified inflammatory responses in multiple organs that were significantly associated with contaminant exposure. These findings indicate that pollutants may impair sea turtle health, likely through immune suppression.

The analysis of tissue alterations in stranded marine animals represents an effective, low‐cost approach for assessing both animal health and environmental quality. When integrated with contaminant data, these observations enhance our understanding of pollutant impacts within the One Health framework, which recognizes the interdependence of human, animal, and ecosystem health and promotes coordinated actions to prevent diseases, protect biodiversity, and improve environmental and public health.

Further research is needed to clarify the toxicological effects of these contaminants and to determine the potential contribution of parasitic infections. Our results also provide valuable insight into pollution levels in southern Brazil, contributing to the goals of the United Nations Ocean Decade—a global initiative aimed at reversing the decline in ocean health through knowledge generation, sustainable development, and informed public policies. Overall, this study advances baseline information for the southern Atlantic region and supports future biomonitoring and contamination assessment efforts.

## Author Contributions

**Camila Roberta da Silva Ribeiro:** investigation, methodology, writing (original draft). **Andressa Maria Rorato Nascimento de Matos:** investigation, methodology. **Eloiza Teles Caldart:** formal analysis, review and editing. **Liana Rosa:** methodology, validation. **Camila Domit:** review and editing. **Guilherme dos Santos Lima:** resources, validation. **Ana Paula Frederico Rodrigues Loureiro Bracarense:** conceptualization supervision, funding acquisition, review and editing.

## Funding

This work was supported by Conselho Nacional de Desenvolvimento Científico e Tecnológico and Coordenação de Aperfeiçoamento de Pessoal de Nível Superior.

## Conflicts of Interest

The authors declare no conflicts of interest.

## Data Availability

The data that support the findings of this study are available from the corresponding author upon reasonable request.
